# Case Report: Rapidly Progressive Interstitial Lung Disease in A Pregnant Patient With Anti-Melanoma Differentiation-Associated Gene 5 Antibody-Positive Dermatomyositis

**DOI:** 10.3389/fimmu.2021.625495

**Published:** 2021-02-25

**Authors:** Cuihong Chen, Yulan Chen, Qin Huang, Qiu Hu, Xiaoping Hong

**Affiliations:** Department of Rheumatology and Immunology, Shenzhen People’s Hospital, The Second Clinical Medical College of Jinan University, The First Affiliated Hospital of Southern University of Science and Technology, Shenzhen, China

**Keywords:** clinically amyopathic dermatomyositis, anti-melanoma differentiation-associated gene 5, rapidly progressive interstitial lung disease, pregnancy, treatment

## Abstract

Dermatomyositis occurs extremely rarely during pregnancy. A number of studies in the published literature have documented how the outcome of pregnancy is poor for both mother and fetus. The present case study reports on a patient who was diagnosed with clinically amyopathic dermatomyositis complicated by interstitial lung disease during pregnancy, and was successfully treated with a combined immunosuppressant regimen. To the best of the authors’ knowledge, this is the first case study detailing how a pregnant woman with clinically amyopathic dermatomyositis with positive anti-melanoma differentiation-associated gene 5 antibody achieved complete remission after early intervention of combined immunosuppressive therapy without residual pulmonary interstitial changes.

## Introduction

Dermatomyositis (DM) is a spectrum of muscle myalgia and weakness, with typical skin manifestations, including heliotrope rash and Gottron’s sign. Clinically amyopathic dermatomyositis (CADM) is an independent spectrum of classic DM with typical skin lesions and minimal or absent muscle disease (1). It has been reported that the prevalence of CADM in all patients with dermatomyositis is 5%–20% (2). Rapidly progressive interstitial lung disease (RP-ILD) is common in CADM patients with positive anti-melanoma differentiation-associated gene 5 (MDA5) antibody (3), which tends to be treatment-refractory with a poor prognosis (4). The onset of CADM during pregnancy is extremely rare and the outcome of pregnancy in these patients is poor for both mother and fetus, including maternal and fetal death, as well as prematurity (5, 6). The present case study reports on a pregnant woman with CADM with the positive anti-MDA5 antibody, who developed RP-ILD during her first 7 weeks of gestation. She was successfully treated with combined immunosuppressive therapy without residual pulmonary interstitial changes.

## Case Presentation

**Figure 1:**
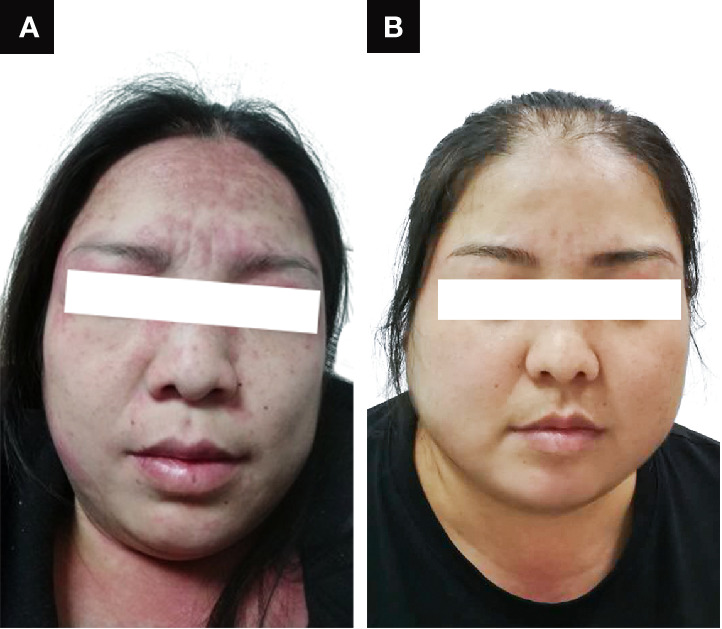
Clinical presentation of the patient **(A)** on admission and **(B)** after recovery.

A 38-year-old woman with a 7-week pregnancy was admitted to our hospital with complaints of dry cough, dyspnea on exertion, and a rash over the face, neck, and dorsum of the hands for 23 days and polyarthritis for 2 days (**Figure 1A**). Prior to admission, she was treated with loratadine without resolution, and her symptoms gradually worsened. Her past medical history was unremarkable.

Physical examination on admission revealed a high body mass index of 33.1, heliotrope rash, Gottron’s papules and fine crackles audible bilaterally in the lower lung fields. No signs of muscle weakness or pain were present. The patient had tachycardia (113 beats/min) with normal oxygen saturation. Laboratory findings revealed the levels of creatine phosphokinase (CK; reference range, 25–192) to be 171 U/l, lactate dehydrogenase (LDH; reference range, 110–240) to be 441 U/l, alanine transaminase (AST; reference range, 0–40) to be 50 U/l, C-reactive protein (CRP; reference range, <5) to be 30.94 mg/l, erythrocyte sedimentation rate (ESR, reference range, 0–20) to be 53 mm/h, and ferritin (reference range, 11.0–306.8) to be 167.3 ng/ml. The main laboratory results are shown in **Table 1**. No evidence was identified that may have suggested infection or malignancy, and therefore CADM was suspected in this patient. She was treated with 24 mg/day of oral methylprednisolone for 3 days, along with 400 mg/day of hydroxychloroquine in her first week of admission. However, the patient’s respiratory condition continued to worsen with percutaneous blood oxygen saturation decreasing to 88% under use of low- flow nasal cannula oxygen and therefore she was given oxygen by way of medium-flow mask oxygen. Moreover, she was unable to complete the pulmonary function test due to the rapid deterioration of the respiratory status.

**Table 1:** Laboratory findings on admission.

Parameter	Value	Reference range
Leukocytes (10^9^/L)	3.6	4–10
Erythrocytes (10^12^/L)	4.76	3.5–5
Hemoglobin (g/L)	129	110–150
Platelets (10^9^/L)	251	100–300
Glucose (mmol/L)	4.89	3.9–6.1
Cholesterol (mmol/L)	3.46	3.4–6.5
HDL (mmol/L)	0.94	0.9–1.91
LDL (mmol/L)	1.69	2.08–4.14
AST (U/L)	50	0–40
ALT (U/L)	48	0–45
ALP (U/L)	61	15–121
CK (U/L)	171	25–192
LDH (U/L)	441	110–240
Urea nitrogen (mmol/L)	2.79	2.5–7.5
Creatinie (µmol/L)	64	44–133
IgG (g/L)	10.21	8–18
Ferritin level (µg/L)	167.3	11.0–306.8
24-h urine protein (g/24 h)	0.216	0.028–0.141
ESR (mm/h)	53	0–20
CRP (mg/L)	30.94	<5
PCT (ng/ml)	<0.05	<0.05
C3 (g/L)	1.28	0.80–1.81
C4 (g/L)	0.32	0.15–0.57
Coombs’ test	Negative	Negative
RF (IU/mL)	7.3	<25
Anti-CCP	Negative	Negative
cANCA	Negative	Negative
anti-PR3	Negative	Negative
pANCA	Negative	Negative
anti-MPO	Negative	Negative
ANA	Negative	Negative
Anti-centromere	Negative	Negative
Anti-dsDNA	Negative	Negative
Anti-SS-A	Negative	Negative
Anti-SS-B	Negative	Negative
Anti-MDA5	Strongly positive	Negative
Anti-tRNA synthase autoantibody panel	Negative	Negative

HDL, high-density lipoprotein; LDL, low density lipoprotein; AST, alanine transaminase; ALT, aspartate aminotransferase; ALP, alkaline phosphatase; CK, creatine phosphokinase; LDH, lactic dehydrogenase; ESR, erythrocyte sedimentation rate; CRP, C-reactive protein; PCT, procalcitonin; RF, rheumatoid factor; C3, complement C3; C4, complement C4; Anti-MDA5, anti-melanoma differentiation-associated gene 5. Anti-CCP, anti-cyclic citrullinated peptide antibody; cANCA, cytoplasmic-staining anti-neutrophil cytoplasm antibody; pANCA, perinuclear-staining anti-neutrophil cytoplasm antibody; Anti-MPO, anti-myeloperoxidase antibody; Anti-PR3, anti-proteinase 3 antibody; ANA, anti-nuclear antibodies; Anti-dsDNA, anti-double-stranded (ds) DNA antibody; Anti-SS-A, anti-Sjögren's syndrome antigen A antibody; Anti-SS-B, anti-Sjögren's syndrome antigen B antibody.

**Figure 2:**
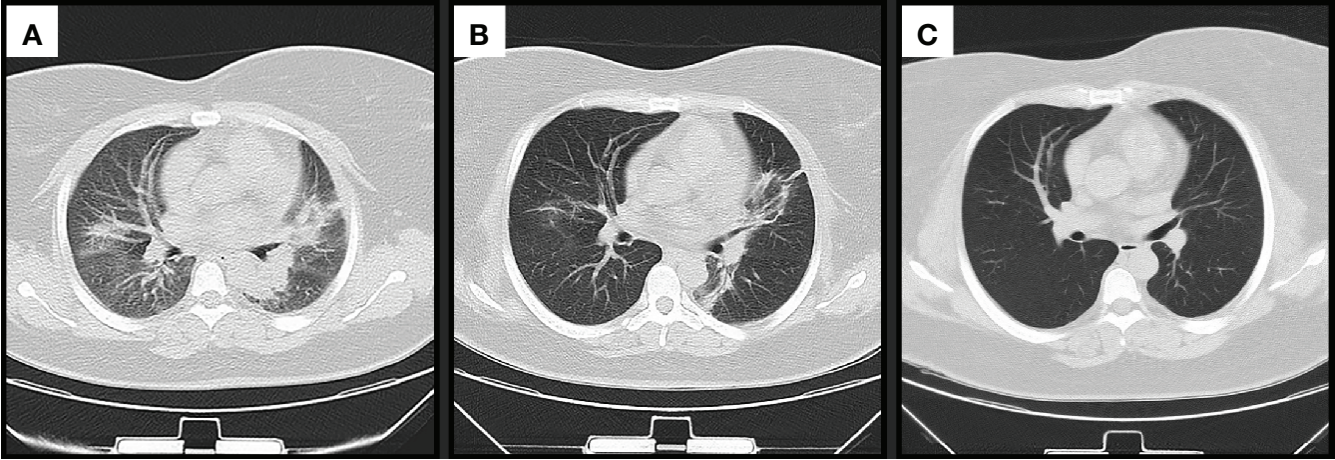
Changes in chest computed tomography scan findings **(A)** on admission, **(B)** before discharge and **(C)** after recovery.

Further tests revealed that her anti-MDA5 antibody was positive. The high-resolution computed tomography (HRCT) scan on day 7 revealed reticular shadows, patchy ground glass opacities and inflammation in both lungs (**Figure 2A**). On the basis of these findings, a diagnosis of anti-MDA5 positive CADM with RP-ILD was made. According to the results of a multi-disciplinary discussion, on day 10, the patient was treated with combination therapy including methylprednisolone (1.0 mg/kg/day, that is, 80 mg/day) accompanied by oral cyclosporine (100 mg twice a day) and intravenous cyclophosphamide (IVCY, 400 mg/week) following high dose pulsed methylprednisolone at 500 mg/day for 3 days and intravenous immunoglobulin (IVIG, 20 g/day for 3 days). Considering her worsening condition, artificial abortion was conducted on day 10 with her permission. The respiratory condition of the patient improved gradually. A chest CT scan performed on day 21 of hospitalization revealed a patchy density shadow that had significantly decreased compared with its appearance previously (**Figure 2B** **)**. The patient was discharged while being treated with methylprednisolone at 80 mg/day, cyclosporine at 200 mg/day, IVCY at 400 mg/2 weeks.

**Figure 3:**
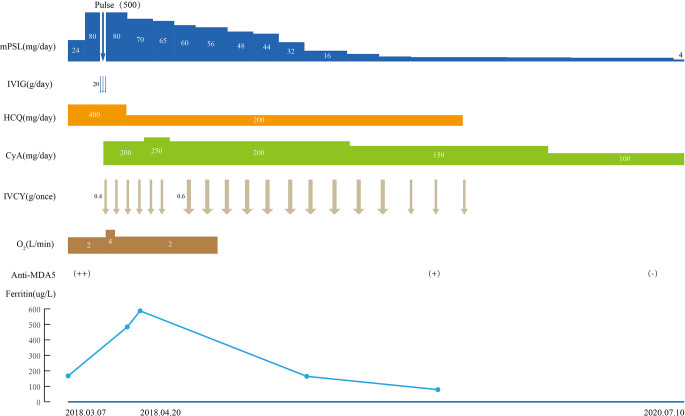
Clinical course of the patient. mPSL, methylprednisolone; Pulse: intravenous methylprednisolone pulse therapy (500 mg/d×3 days); IVIG, intravenous immunoglobulin; HCQ, hydroxychloroquine; CyA, cyclosporine A; IVCY, intravenous cyclophosphamide; O_2_, oxygen; Anti-MDA5, anti-melanoma differentiation-associated gene 5 antibody; ++, strongly positive; +, positive; −, negative.

Because of the significant improvement in her respiratory condition, we decided to taper the methylprednisolone and IVCY dose gradually. During the follow up, the patient achieved complete remission, her rash disappeared (**Figure 1B**) and the chest CT scan revealed that the patient had returned to the normal state after treatment for 10 months (**Figure 2C** **)**. IVCY treatment was discontinued after treatment for 14 months, and anti-MDA5 antibody was negative after treatment for 24 months. At the time of writing this report, the patient was being treated with 4 mg/day methylprednisolone and 75 mg/day cyclosporine. The clinical course of the patient is shown in **Figure 3**.

## Discussion

Inflammatory myopathies are a spectrum of systemic immune-mediated disorders characterized by muscle inflammation, and affecting extramuscular organs, including skin, joints, and lungs. It is widely recognized that there are five main types of inflammatory myopathies: DM, polymyositis (PM), overlap myositis (including anti-synthetase syndrome (ASS)), inclusion-body myositis, and immune-mediated necrotizing myopathy (7, 8). RP-ILD was mainly defined as progressive dyspnea, or according to HRCT findings secondary to ILD within 3 months after the onset of respiratory symptoms (9, 10). RP-ILD often occurs as a complication of CADM, which usually portends poor prognosis with reported mortality rates in the first year as high as 60% (11). Anti-MDA5 antibodies were first identified by Sato et al. in 2005 in Japanese patients with CADM (12). Since that discovery, anti-MDA5 antibodies have predominantly been found in cases of CADM (13). Mortality rates ranged from 36%–46% in anti-MDA5 positive patients with CADM or DM (14). Anti-MDA5 antibodies have also been associated with RP-ILD, as identified in 39%–100% of patients with RP-ILD in Asian cohorts (15). The cumulative 6-month survival rate was reported to be about 50% for RP-ILD patients with the anti-MDA5 antibody (16). Hence, anti-MDA5 antibodies can be a useful predictor for the complication of RP-ILD in patients with CADM. In the present case study, a diagnosis of anti-MDA5 positive CADM with RP-ILD was made as the patient experienced respiratory symptoms from the beginning, and her respiratory status deteriorated rapidly within one month. It has been suggested that immunosuppressants should be used early in patients with RP-ILD, since the damage caused to the pulmonary tissue may be irreversible once CADM is complicated by RP-ILD, which may result in the patient being irresponsive to combination immunosuppressive therapy, leading to death after only a few months (17). However, the use of immunosuppressants may not ensure rapid remission of the patient’s condition (18). It was reported that combination immunosuppressive therapy consisting of high-dose corticosteroids, cyclosporine and intravenous immunoglobulin might be effective for CADM patients with RP-ILD (16). Moreover, previous studies have also shown that patients with RP-ILD or respiratory failure may benefit from the use of basiliximab, mycophenolate mofetil or cyclophosphamide (19, 20).

The onset of DM during pregnancy is a rare event. It has been reported that one in 173 female patients with DM/PM experienced disease onset during pregnancy (21). Pregnancy outcomes are closely associated with the disease activity during pregnancy. Patients with quiescent disease before pregnancy have good pregnancy outcomes. By contrast, a pre-existing active condition or onset of DM during pregnancy leads to a high frequency of premature delivery and fetal death (21).

There are several factors that may explain the trigger for development of DM during pregnancy, such as changes in maternal hormonal status, exposure of the mother to fetal antigens, and the reactivation of certain viruses due to pregnancy (6). In addition, a high body mass index was noticed in the patient. Previous studies have revealed that obesity is a metabolic disease which may lead to the activation of the immune system and a consequently worse prognosis (22). Moreover, the obese are prone to respiratory failure even with mild pulmonary challenge (23). Hence, the rapid deterioration of the respiratory status in this patient may be partially explained by her high body mass index.

In view of these considerations, the onset of CADM during pregnancy is a problematic issue to be resolved for both physicians and patients. Treatment regimen should consider the safety of both the mother and the fetus, which requires individualized therapy. Glucocorticoids are the first line treatment in pregnant patients with DM (24). In certain rare cases, the use of glucocorticoids has been demonstrated to lead to a good outcome (6). However, certain patients have been shown to be non-responsive or intolerant to glucocorticoids (24). A previous study illustrated that gestational exposure to glucocorticoids led to a slight increase in the risk of premature birth and fetal oral cleft (25). Efficacy and safety of IVIG during pregnancy has been well documented in DM, especially for refractory cases (24, 25). In a previously published case report, short term remission was achieved following treatment with IVIG (4.5 g for 3 consecutive days) (25).

Examples of clinical case studies for treating pregnant patients with DM or PM, especially CADM, are relatively rare, and case studies of a similar nature that we were able to identify are shown in supplementary material (18, 26–28). Tomohiro et al (18). described the case of a 33-year-old pregnant woman who developed progressive interstitial pneumonia (IP) complicated by anti Jo-1 positive ASS at 28 weeks of gestation. At 30 weeks of her gestation, since the neonate could be treated at the neonatal intensive care unit after delivery, an emergency cesarean section was performed. The patient was eventually successfully treated with a combination of immunosuppressive therapy including intravenous methylprednisolone pulse therapy (1 g/day) and IVCY and the use of high flow nasal cannula oxygen therapy without intubation. In another case report, a pregnant woman at 16 weeks of gestation had developed IP preceding anti Jo-1 positive ASS, and was treated with a combination of steroid pulse therapy and tacrolimus. The fetus did not survive since it was too small, and the maternal condition deteriorated (26). To the best of our knowledge, the present case study is the first reported case of a patient having been diagnosed with anti-MDA5-positive RP-ILD complicated with CADM. In our case, artificial abortion was conducted because the maternal condition was unstable. The influences of high dose steroids and the CT scan were also taken into consideration. After combining steroid pulse and immunosuppressive therapy, the maternal condition gradually improved and our patient had complete remission without residual pulmonary interstitial changes. In anti-MDA5-positive patients with CADM, the CT scans usually show ground-glass opacities and bilateral subpleural reticular opacities, predominantly in the lower lungs, which would improve substantially or become stable after combination immunosuppressive therapy (29, 30). The present report describes a pregnant woman with CADM with positive anti-MDA5 antibody, who developed RP-ILD and subsequently achieved complete remission without residual pulmonary interstitial changes after treatment, which has been rarely reported in the previous literature. It is difficult to save the fetal lives in CADM patients with anti-MDA5 antibody. Because it was dispensable to use combination of high dose corticosteroids and immunosuppressive agents including IVCY in CADM patients even if they were in pregnancy.

Certain biological parameters have been evaluated for their ability to predict the disease activity, occurrence, and outcomes of patients with CADM. As mentioned above, both anti-MDA5 antibodies and RP-ILD are crucial predictors. Furthermore, LDH, Krebs von den Lungen 6 (KL-6), serum surfactant protein D (SP-D), ferritin level, and the HRCT imaging score are also associated with prognosis of the disease (14, 31). In this case, the patient’s ferritin level was elevated when her respiratory condition continued to worsen, which also suggested a poor prognosis in our patient. Recently, Lian et al. established a simple and practical score model to predict the prognosis of patients with ADM-ILD (31). According to this model, our patient was at least in the medium risk group, with a score of 5 (three points for anti-MDA5 antibodies, and two points for RP-ILD). Despite the fact that not all patients with this antibody develop lethal ILD, one possible explanation may be that all of the patients who have this particular antibody may undergo an early stage of disease, with only a skin rash or skin rash accompanied by arthralgia. They subsequently develop asymptomatic ILD, which progresses to RP-ILD if left untreated (32). The main limitation of the current study is that this is a case report, and several cases of CADM and DM developing during pregnancy have been previously described. However, we have made a comprehensive review, and to be best of our knowledge, this is the first case study depicting a pregnant patient with anti-MDA5-positive RP-ILD complicated with CADM. Notably, the patient finally achieved complete remission without residual pulmonary interstitial changes, indicating the importance of early intervention with combined immunosuppressive therapy in such patients.

## Concluding Remarks

The present case study has reported on a case of a 38-year-old pregnant woman at 7 weeks of gestation who developed RP-ILD due to CADM with positive anti-MDA5 antibody. She achieved complete remission following early intervention of combination immunosuppressive therapy without residual pulmonary interstitial changes.

## Data Availability Statement

The raw data supporting the conclusions of this article will be made available by the authors, without undue reservation.

## Ethics Statement

The studies involving human participants were reviewed and approved by Medical Ethics Committee of Shenzhen People’s Hospital. The patients/participants provided their written informed consent to participate in this study. Written informed consent was obtained from the individual(s) for the publication of any potentially identifiable images or data included in this article.

## Author Contributions

CC and YC summarized the case, reviewed the literature, and drafted the manuscript. QinH and QiuH drafted the manuscript. XH reviewed and summarized the case. All authors contributed to the article and approved the submitted version.

## Funding

The research is supported by Shenzhen Key Medical Discipline Construction Fund (no. SZXK011), Shenzhen Health Plan Committee Research Foundation (no. SZXJ2018021), Shenzhen Science and Technology Plan Program (no. JCYJ20190807144418845), and Sanming Project of Medicine in Shenzhen (no. SYJY201901).

## Conflict of Interest

The authors declare that the research was conducted in the absence of any commercial or financial relationships that could be construed as a potential conflict of interest.
