# Supplementary material for: Case Report: Rapidly Progressive Interstitial Lung Disease in A Pregnant Patient With Anti-Melanoma Differentiation-Associated Gene 5 Antibody-Positive Dermatomyositis
Source: Front Immunol. 2021 Feb 25;12:625495. doi: 10.3389/fimmu.2021.625495 (PMC7947909; doi:10.3389/fimmu.2021.625495)
Supplement: Supplementary file 1 [file Table_1.docx]

**Supplemental Table 1 Pregnant patients with inflammatory myopathies and interstitial lung disease reported in the literature**

| Ref | Diagnoses | Gestation | Antibodies | Treatment | Outcome |
| --- | --- | --- | --- | --- | --- |
| 26 | PM/IP | 16 | Jo-1 | Prednisolone; tacrolimus; abortion; cyclophosphamide pulse therapy | Abortion/IP improved |
| 18 | CADM/IP | 28 | Jo-1 | Methylprednisolone pulse therapy; HFNC; cesarean section; cyclosporin; cyclophosphamide pulse therapy | Cesarean/interstitial shadows disappeared |
| 27 | PM/ILD | 8 | Jo-1 | Prednisolone; cyclosporine | Delivery/stable condition |
| 28 | CADM/ILD | 10 | EJ | Prednisolone | Delivery/improved |

Ref, references; PM, polymyositis; IP, interstitial pneumonia; ILD, interstitial lung disease; CADM, clinically amyopathic dermatomyositis; HFNC, high ﬂow nasal cannula oxygen therapy.
